# The meaning of manageable neuropathic pain after SCI

**DOI:** 10.3389/fpain.2025.1540395

**Published:** 2025-06-11

**Authors:** Marlon L. Wong, Kimberly D. Anderson, Kathryn E. Roach, Linda Robayo, Nicholas P. Cherup, Roberta Vastano, Gabriel Fernandez, Eva Widerström-Noga

**Affiliations:** ^1^ Department of Physical Therapy, Miller School of Medicine, University of Miami, Coral Gables, FL, United States; ^2^ Institute of Functional Restoration, MetroHealth System, Case Western Reserve University School of Medicine, Cleveland, OH, United States; ^3^ Miami Project to Cure Paralysis, Miller School of Medicine, University of Miami, Miami, FL, United States

**Keywords:** neuropathic pain, spinal cord injury, manageable pain, qualitative research, mixed-methods research

## Abstract

**Introduction:**

Chronic neuropathic pain (NP) is a prevalent and debilitating condition among individuals with spinal cord injury (SCI). Complete pain relief is often unattainable, making the concept of “manageable pain” a critical focus for improving quality of life. This study aims to elucidate the meaning of manageable pain for individuals with chronic NP post-SCI.

**Methods:**

A mixed-methods approach was employed, involving qualitative interviews and quantitative assessments with 36 participants experiencing moderate to severe NP.

**Results:**

The qualitative data revealed three major themes: Manageable Pain, Unmanageable Pain, and Ways to Control Pain. Manageable pain was characterized by its moderate intensity, predictability, and minimal interference with daily activities. In contrast, unmanageable pain was associated with significant emotional distress, activity hindrance, and inability to control the pain. Participants used a variety of techniques to control pain, including cognitive/emotional coping strategies, medication, and physical activity. Most participants used a multimodal approach that was severity and situation dependent.

**Discussion:**

These findings underscore the multifaceted nature of pain management and the importance of individualized approaches that consider both pain acceptance and coping strategies. This study provides valuable insights into the personal experiences of NP in people with SCI and their perspectives on the meaning of manageable pain. These findings highlight the need for comprehensive pain management strategies that enhance daily functioning and overall well-being.

## Introduction

Most people with spinal cord injury (SCI) suffer from chronic neuropathic pain (NP), and complete pain relief is often an unrealistic goal (1, 2). A more realistic goal is to reduce pain's impact on quality of life and to make pain “manageable”. Manageable or tolerable pain is a construct that is not specific to NP after SCI, and research in other populations suggests that manageable pain is pain that allows the performance of daily activities or “getting something done” (3). Manageable pain may also be associated with lower levels of affective distress and less pain interference with social activities.

The concept of manageable pain has been studied using a quantitative approach in heterogeneous pain populations, including people with chronic knee osteoarthritis, rheumatoid arthritis, limb amputation, cancer, and low back pain (4–6). For instance, manageable pain was established via cut points on the numeric pain rating scale (NPRS) that correlate with moderate functional impairment (4–6). Specifically, scores between 4 and 6 (from 0 to 10) correlates with moderate functional impairment, which individuals typically deem as intolerable pain. Although this approach provides valuable information that can be used in analyses for quantitative studies, there is often a non-linear relationship between pain severity and interference with function (5, 7–9). This is because many other factors may influence pain interference (e.g., personal coping skills, psychological strength, qualitative nature of pain, and presence of evoked pain). Additionally, other studies suggest that traditional pain scales, like the NPRS, may not be interpreted consistently across patients. Instead, using simpler binary questions about pain acceptability may offer clearer insights, though a multidimensional approach to pain assessment remains ideal for understanding what constitutes acceptable pain (3, 10, 11). Although interesting, the overall findings from these studies do not provide insight into the *meaning* of manageable pain for individuals within these populations.

We have only identified one qualitative study that directly investigated the meaning of manageable pain. Zelman et al. (3) conducted focus groups of people with pain associated with metastatic cancer, osteoarthritis, and low back pain to identify the daily goals of people who use analgesic medications for controlling persistent pain. Interestingly, the participants objected to the term “acceptable day of pain” and preferred the terms “manageable” or “tolerable”. Zelman identified five key themes: in order of importance, participants stated that on a desirable day, (1) their medication takes the edge off the pain, (2) increased function is possible, (3) social engagement is desired, (4) there is sufficient nighttime rest, and (5) they have reduced negative affect. These themes provide important insight into the meaning of manageable pain, suggesting that the impact of manageable pain is not merely the result of controlling the intensity or unpleasantness of symptoms, but rather the controlling of pain to the point of approximating an unhindered life. To date, little is known about what manageable pain means, specifically to people with chronic NP after SCI.

Given the unique characteristics of people with NP after SCI, caution should be used when extrapolating Zelman et al.'s findings to this population. For example, it is known that many people with NP after SCI are doing relatively well despite having severe pain (12). Moreover, Zelman et al.'s findings were specific to “a day of manageable pain” *with medication use*. However, it is known that people with chronic NP after SCI do not always use medication for pain management and that they often engage in a variety of pain management strategies (12, 13). In fact, a previous study found that those with moderate NP after SCI used multiple approaches to manage their pain, and they used less medication than those with severe pain due to concerns about side effects and addiction (12). Thus, the purpose of this study was to examine what the term “manageable pain” means specifically to people with chronic NP after SCI. Further, we explored the differences in pain-related and psychosocial impact between those who experience manageable pain every day and those who do not have manageable pain every day.

## Methods

This study was part of a larger mixed methods study regarding participants' perspectives on a novel multimodal pain intervention program. For the purposes of the present article, qualitative interview data concerning the concept of manageable pain and pain evaluations were analyzed at the first interview in 36 participants with moderate to severe NP associated with SCI. The study adhered to the principles of the Declaration of Helsinki and was approved by the Institutional Review Board of the University of Miami Miller School of Medicine.

### Participants

Participants (*n* = 36) were recruited via flyers posted at the University of Miami clinics and at the Miami Project to Cure Paralysis. We also recruited participants from the Miami Project SCI volunteer research database. Men and women aged 18–70, with traumatic incomplete or complete SCI (incomplete injuries retain some level of function, whereas complete injuries result in total loss of function below the injury site) with moderate NP intensity or above (*N*PRS >3/10), were invited to participate. Potential participants were excluded if they reported a history of systemic illness (e.g., cardiovascular disease, multiple sclerosis, rheumatoid arthritis, cancer), severe depression (BDI-II >29), body mass index (BMI) >35, or scored above the threshold for unhealthy alcohol (AUDIT >10) or drug (DAST-10 >6) use within the past year.

### Measures

#### Screening

##### Beck depression inventory, 2nd edition (BDI-II)

The BDI-II is a 21-item questionnaire for the assessment of depressive symptoms consistent with the DSM-IV. Common depressive symptoms over the past two weeks are rated on a 4-point scale from 0 to 3, with the overall score ranging from 0 to 63. Range of depression: 0–13 minimal, 14–19 mild, 20–28 moderate, and 29–63 severe. The internal consistency and reliability of the BDI-II among those with chronic pain conditions has been previously established.

##### Alcohol use disorder identification test (AUDIT)

The AUDIT consists of 10 items about alcohol use, alcohol dependence symptoms, and alcohol-related problems over the past year.

##### Drug abuse screening test (DAST-10)

The DAST-10 is designed to detect drug-related problems over the past year. The DAST includes 10 items rated on a yes/no binary. The DAST-10 is psychometrically consistent and reliable among various populations.

#### Qualitative data

Semi-structured interviews were conducted after receiving 1 month of educational sessions on pain science and management strategies, again after receiving an additional 6-weeks of guided exercise and visual illusion training, and a third time 1 month after completion of all intervention activities. Each interview was conducted by one investigator (MW) via Zoom. For the purposes of this manuscript, we only focus on the responses to the first question posed during the first interview: “What is manageable pain to you?” For participants who had difficulty responding to the question, we followed up with the opposite, “What is unmanageable pain to you?”

Interviews were audio recorded and transcribed verbatim by an independent transcribing service. A qualitative content analysis was performed by two independent researchers (EW and KA) who reviewed and described the information using NVIVO (software headquartered in Lumivero, Denver, CO). The study staff also completed quality control procedures to ensure data accuracy. First, major themes were independently coded by two investigators (EW and KA). A third investigator (MW) was added to the analysis process to interrogate the transcripts for sub-ordinate themes. The primary intent was to describe rather than to generate new theories, and the qualitative approach was designed to allow the researchers to adapt their methods to fit the specific needs of their study as the project unfolded, rather than adhering to a specific qualitative tradition. Thus, a generic qualitative research design (14), from an interpretivist paradigm, was employed for this project. Based on our prior studies, we anticipated achieving saturation within 35 participants, and no new themes emerged after 30 SCI participants. Therefore, we completed our interviews with 36 individuals with SCI-associated NP. The identified themes were then discussed in study team meetings and revised if needed.

Investigators EW and KA were both senior investigators with extensive experience in qualitative and quantitative research focused on pain in the SCI population. Investigator KA also has a SCI, providing her with a unique perspective in this work. MW is a physical therapist with extensive clinical experience in pain management and some previous experience with SCI research. These investigators had minimal contact with the study participants outside of the interviews, and they did not have prior relationships with any of the participants.

#### Quantitative data

To further evaluate participants' pain, psychosocial factors, and medication use, the following assessments were administered:

##### Days with manageable pain

This item specifies the total number of days with manageable pain during the last 7 days, including today, and the response categories range from 0 = none to 7 = seven days. This item is part of the International SCI Extended dataset (15). Because the perception of days with manageable pain is likely to change during the course of a study, we used assessment and interview data collected at the first interview for all analyses.

##### The international spinal cord injury pain basic data set

This tool was primarily developed to provide clinically relevant information concerning SCI-related pain that could be collected by healthcare professionals, and it has served as a basic pain measure in clinical research and clinical registers (16–18). Four items were extracted from this tool for this study: (1) “Number of days with manageable/tolerable pain in the last 7 days including today.”; (2) “In general, how much has pain interfered with your day-to-day activities in the last week?” (interference with activities); (3) “In general, how much has pain interfered with your overall mood in the last week?” (interference with mood); (4) “In general, how much has pain interfered with your ability to get a good night's sleep in the last week?” (interference with sleep); and (5) “Overall, how hard is it for you to deal with your pain?” (hard to deal with pain). For days with manageable pain, possible scores ranged from 0 to 7, and for all other items possible scores ranged from 0 = “No interference” or “not hard at all” to a maximum of 10 = “Extreme interference” or “extremely hard”.

##### Multidimensional pain inventory: SCI version (MPI)

The West Haven-Yale MPI (19) is a comprehensive instrument designed to assess a range of self-reported behavioral and psychosocial factors associated with chronic pain syndromes. The total scale consists of 50 items, but only the following subscales were used, Pain Severity (MPI-PS, 3-items), Life Interference (MPI-LI, 8-items), Life control (MPI-LC, 3-items), and Affective distress (MPI-AD, 3-items). All items are scored on a 7-point Likert scale (0–6), and the subscale score is reported as the mean of component items.

##### Neuropathic pain symptom inventory (NPSI)

The NPSI is one of the most widely used tools for characterizing NP symptom severity (20, 21), and it is comprised of 10 items scored on 0–10 Numerical rating scales, and that assess dimensions of NP (burning spontaneous pain, pressing spontaneous pain, paroxysmal pain, evoked pain, and paresthesia/dysesthesia). NPSI total score ranges from 0 to 100, with higher scores indicating worse NP severity. The NPSI demonstrated good psychometric properties in a cohort of people with NP after SCI (22).

### Quantitative data analyses

Descriptive statistics (i.e., means, medians, frequencies, percentages, and standard deviations) were used for all variables, when appropriate. Spearman's rank correlation analysis was used to explore the associations between the number of days per week with manageable pain (M-pain days) and the MPI-SCI subscales and interference scores. All statistical analyses were conducted using Statistical Package for the Social Sciences (SPSS) v28 (IBM Corp., Armonk, NY), and figures were rendered using GraphPad Prism v9.3.1 (GraphPad Software, La Jolla, CA). Results were considered significant if values met the *a priori* threshold set at *p* ≤ 0.05.

## Results

### Demographic and injury-related characteristics

The demographic characteristics of the sample are described in Table 1. The mean age (standard deviation) of the sample was 42.3 (14.0) years, with ages ranging from 19 to 74. Most participants identified as male (75%) and single (69.4%). There was a wide range of education levels, with over 58% having at least some college experience or holding an associate degree. Additionally, a little more than half of the participants had tetraplegia (55.6%), there was representation across all levels of the American Spinal Injury Association Impairment Scale [(AIS), levels A–D], and the average time since injury was 10 years for the entire group.

**Table 1:** Demographic and pain characteristics.

Characteristic		Total *N* = 36
Age (mean, SD)		42.3 (14.0)
Sex (*n*, %)	Male	27 (75)
	Female	9 (25)
Ethnicity (*n*, %)	White non-Hispanic	8 (22)
	Hispanic	13 (36)
	African American	8 (22)
	Asian	1 (3)
	Other	6 (17)
Education (*n*, %)	Less than high school	4 (11)
	High school	9 (25)
	AA or some college	14 (39)
	Bachelor	4 (11)
	Advanced degree	3 (8)
	Other	1 (3)
Multidimensional pain inventory (MPI) subscales (mean, SD)	Pain severity (PS)	4.0 (1.7)
	Life interference (LI)	2.9 (1.8)
	Life control (LC)	3.9 (1.4)
	Affective distress (AD)	2.4 (1.5)
Neuropathic pain symptom inventory (NPSI) (mean, SD)		42.1 (22.5)
The international spinal cord injury pain basic data set (mean, SD)	Interference with activities	4.4 (3.7)
	Interference with mood	4.1 (3.2)
	Interference with sleep	4.9 (3.5)
	Difficulty dealing with pain	4.8 (3.1)

### Qualitative data

Some participants had clear interpretations of manageable pain, while others found the concept nebulous and had difficulty describing manageable pain. Those who had difficulty describing manageable pain often chose to define what made pain unmanageable or described how they controlled their pain. Thus, three major themes emerged: (1) Manageable Pain, (2) Unmanageable Pain, and (3) Ways to Control Pain (Table 2). Within each of these major themes, key subthemes were also identified, and the themes and subthemes collectively provide a detailed understanding of how these individuals conceptualize the manageability of their pain. For many participants several of the subthemes were expressed, thus demonstrating the multifactorial nature of how they defined manageable pain.

**Table 2:** Major themes and subthemes.

Major themes:	Manageable pain	Unmanageable pain	Ways to control pain
Sub-themes:	• Pain characteristics • Pain that can be controlled, managed, and dealt with • Pain that can be ignored or tolerated • Pain that does not interfere with daily life activities	• Affects mood • Hinders activity • Cannot be controlled	• Ignoring pain, using distraction • Prescription and non-prescription medication use • Using multiple approaches • Severity and situation dependent • Staying active, exercise

The theme Manageable Pain included four subthemes: (1) Manageable pain characteristics, (2) Pain that can be controlled, (3) Pain that can be ignored or tolerated, and (4) Pain that does is not interfere with daily life activities.

#### Manageable pain characteristics

The characteristics of the pain (i.e., the intensity and temporal pattern) were a defining feature of manageable pain for some. Several participants defined manageable pain as being below a 4 or 5 on an 11-point NPRS. “*Ideally, it would be zero pain, but that's pretty hard. That's pretty hard to get. Once in a while, I do get that. I think it's mostly when I'm being distracted, but manageable pain. Let's see. I think probably if you're using that scale, I think a four or five would be manageable.*” (Participant 12). Manageable pain was also described in terms of the temporal pattern: “*It would be more consistent pain rather than the spikes.*” (Participant 24).

#### Pain that can be controlled

Many participants (*n* = 19) described how being able to do something to reduce the intensity of the pain or take the edge off, was an important feature in making it manageable. As one participant said, “*It's pain that I could somewhat control. A pain that I could possibly take some type of medication or do some type of non-medicated therapy that could actually help alleviate or control the pain to some degree…*” (Participant 25). Another participant described it as “*Pain that you can … what's the word I'm looking for … relieved by either medication or exercise or meditation or any type of form where you can manage it where it's not overwhelming or controlling your life in a negative way.*” (Participant 8). This also highlights the importance of having knowledge of a variety of pain management strategies, and the availability of pain management tools, for many people to achieve manageable pain after SCI.

• “*It's just finding a way to manage pain. Sometimes finding ways like toughening out or, I guess, researching with your pain management to see what can help out or anything like that.*” (Participant 21)
• “*it means being able to manage it, to control it, to take care of your pain when you … Manageable pain to be able to manage it.*” (Participant 17)

#### Pain that can be ignored or tolerated

Many participants described the ability to ignore or tolerate the pain as a defining feature of manageable vs. unmanageable pain. For some, this was described as a struggle:

• “*It's something that I could force myself to deal with for a little period of time.*” (Participant 12)
• “*The ability to forget about it. So it could still be there, but you just aren't consciously thinking about it.*” (Participant 19)

For others, it was described as a process of acceptance:

• “*Just dealing with (it) I got to deal with to get through it.*” (Participant 27)
• “*If it doesn't go away, I have to learn how to live with it.*” (Participant 22)

#### Pain that does not interfere with daily life activities

The impact of pain on movement and activities of daily living was another defining feature of manageable for many participants. This is captured in the following quotes:

• “*It means that I can go out for a little bit throughout the day and that the pain isn't stopping me from living my daily life. I always have pain, but the manageable pain is that I can go to the store for an hour or go sit in the car, or go out and do something like that, then go back home.*” (Participant 10)
• “*Basically that something that you can go through the day without having to say, well cut back anything you have to do.*” (Participant 29)
• “*Pain that isn't bothersome so I can continue living my life. That like it's there, but it's not going to affect my day-to-day…*” (Participant 19)
• “*I could still get up and still do my daily tasks without … Well, I will complain but I'm still doing it. That's manageable.*” (Participant 26)

### Unmanageable pain

Many of the participants experienced some days of unmanageable pain. The theme Unmanageable Pain included three subthemes: (1) affects mood, (2) hinders activity, and (3) cannot be controlled or tolerated.

#### Affects mood

Some participants described high levels of emotional distress associated with unmanageable pain:

• “*I'll tell you, sometimes I want to give up. I have my days where I don't want to be bothered. I have plenty of days where I just sit there and cry. Why do I cry? I don't know. But I just be boo-hoo crying.*” (Participant 04)
• “*Oh, man. I don't want to go through this.* *I would probably rather be dead than deal with something like this. It's killing you. Softly. Maybe not saying anything, but it's killing you. Even if nobody does not see it; who can see it? You're the only one that knows because you don't want to feel it.*” (Participant 16)

#### Hinders activity

Unmanageable pain also evoked vivid descriptions of suffering and inability to move due to the pain:

• “*Yes, especially when I'm going to a dinner or I'm going to a meeting. When I'm going to an expo, when I'm going out with my family, business, one of the main things business is, how's my pain going to be while I'm sitting there in a seven-hour conference?*” (Participant 1)
• “*where I really can't be doing most things, having a hard time getting along with people and not wanting to go out. Just being basically, I guess you could say, incapacitated, disabled from the pain.*” (Participant 35)

#### Cannot be controlled or tolerated

Unmanageable pain was associated with a sense of helplessness and no relief. The sense of lack of control is portrayed in the following quotes:

• “*Pain that really cannot control. For example, if I am in my bedroom, if there is no any other visual or physical activities to do, I'm just by myself, very quiet. In a very quiet environment, I would feel the pain sitting there or in my bed doing nothing. So that's where, unfortunately, I can experience the pain and nothing to do about it.*” (Participant 18)
• “*unmanageable is not being able to control it and it just having a mind of its own like it does…*” (Participant 36)

### Ways to control pain

The final major theme, Ways to Control Pain, included the subthemes (1) ignoring pain, using distraction, (2) using medication, (3) using multiple approaches, (4) severity and situation dependent, and (5) staying active or exercising.

#### Ignoring pain, using distraction

Many of the participants described the process of mentally “blocking out” the pain as a coping mechanism. For most, distraction was the primary method of blocking out the pain:

• “*But I always try to find a positive way of try to distract it from taking advantage of my life and pretty much controlling my life throughout. I know that it's there, but I try to black it out, pretty much.*” (Participant 6)
• “*I put it in the mentality of,* “*Okay, is this pain hurting or is it bothering me?*” *Most of the times, it's just bothering me. It's not something that I cannot, how do I say it? It's not something that I cannot take all the time. Sometimes it gets really bad, but the moment I stop thinking about it or I get busy doing something else, it diminishes, it slows down.*” (Participant 37)
• “*So I just pay attention to how I'm feeling, and just let it pass with activity sometimes…*” (Participant 11)

#### Using medication

Participants used a variety of medications, including anticonvulsants, antidepressants, opioids (i.e., oxycodone), nonsteroidal anti-inflammatory drugs (i.e., diclofenac, acetaminophen, and ibuprofen), and anesthetic patches and ointments. Although the failure of medication to sufficiently alleviate symptoms and the negative effects of pain medications were reported by some participants, others described the use of prescribed medication as an important strategy for their pain management:

• “*But that's a big difference when you don't take a medication than when you do take your medication. And then once you take your medication… It's there. It never goes away, with the medication or whatever. With the medication, it's just a way to cope with it.*” (Participant 6)
• “*taking meds. That's the only way right now that I'm able to control it and go out and do whatever I need to get done. You know, like maybe doctor's appointments, or maybe even going to the rehab center. I have to take a gabapentin and a clonazepam.*” (Participant 28)

Several participants also described self-medicating with cannabis:

• “*I'm accustomed to it, so I just deal with it. I ain't going to lie, I smoke my little weed. Weed helps ease the pain too. It helps a whole hell of a lot.*” (Participant 4)
• “*but I honestly just smoke weed for my pain if it gets to that point. I'm not a person that takes pills or anything of the nature.*” (Participant 11)

#### Using multiple approaches

Most participants described using a multimodal approach to control their pain prior to their participation in the study, with varying forms and combinations of cognitive strategies, drugs, physical agents (i.e., thermal modalities and TENS), and physical activity.

• “*For example, this Saturday was my granddaughter's one year old, but I woke up with tremendous pain, one of the worst, and I had to take some marijuana cream and then I had to take Motrin to see if I could manage my pain. Where I do manage my pain more than when I just told you about it is that I redirect my pain in my thoughts. I just don't think about it. I try to just focus on the event and everything else. I blank out on the pain.*” (Participant 1)
• “*Tools being medication, stretches, exercise, rests, the list could go on, and those tools help when I get in extra pain.*” (Participant 24)
• “*I do a lot of stuff. I do a lot of reading, I do a lot of stretching, I do a lot of exercise. I do a lot of massage, I massage myself. I do a lot of stretching. I do some breathing techniques, meditation.*” (Participant 32)

#### Severity and situation dependent

Participants also modified their pain management approach based on the severity of the pain, with a preference for nonpharmacological approaches when the pain is mild and use of medications when the pain is severe.

• “*If it's really intense pain, then I may have to use a medication or analgesic cream on it. If it's not that bad, it's just a matter of a mind over matter.*” (Participant 8)
• “*Once it starts climbing on the uncomfortable level, I have tried to rub different things, like on my arms, or for example, when it's on my butt, which is usually where I get the other pain, I usually just get off of it from sitting down. I just just get on my bed, lay on my side, just if I can stand or I can do something else, just relieve that pressure essentially*” (Participant 35)
• “*Sometimes exercise. Sometimes medication. Sometimes deep breathing techniques. There are options. Depends on what I'm going through like that, but those are the main things actually.*” (Participant 27)

#### Staying active, exercise

Strategies involving physical movement were the most widely endorsed pain management strategies. Exercise and engaging in physical activity (e.g., sports) were often used as a distraction and coping mechanism even in cases when activity did not provide direct pain relief.

• “*Medication in a workout. I do stay active. Movement for my body, whatever my injuries are and whatever the signals are sent, the more I'm active, more warmed up I am, I can use my walker and push myself… stretching and exercise works better than any other therapy for me.*” (Participant 03)
• “*At the beginning, I have to tolerate the pain that I was having at that moment, still have. But it seems that playing the sport, it really mentally and physically minimize the pain. I've been playing a lot of sports.*” (Participant 18)
• “*when you got the exercise, when you have the exercise, your pain is not the same when you don't have the exercise. Now, when you manage your pain, when you manage for exercise, your pain's coming better now.*” (Participant 20)

### Quantitative data

The mean (standard deviation) for the NPSI score was 42.1 (22.5), indicating that NP symptoms were moderate on average. Participants used a variety of medications to manage pain symptoms, and the most frequently used were anticonvulsants (i.e., gabapentin and pregabalin), which were used by 56% (*n* = 20) of the participants. This is not surprising given that these medications are considered first-line treatments for NP after SCI (23). Other medications reported were antidepressants (11%, *n* = 4), muscle relaxants (25%, *n* = 9), nonsteroidal anti-inflammatory drugs (17%, *n* = 6), and opioids (22%, *n* = 8). Interestingly, Cannabis was used in over 22% (*n* = 8) of cases, suggesting an emerging preference for alternative therapies.

**Figure 1:**
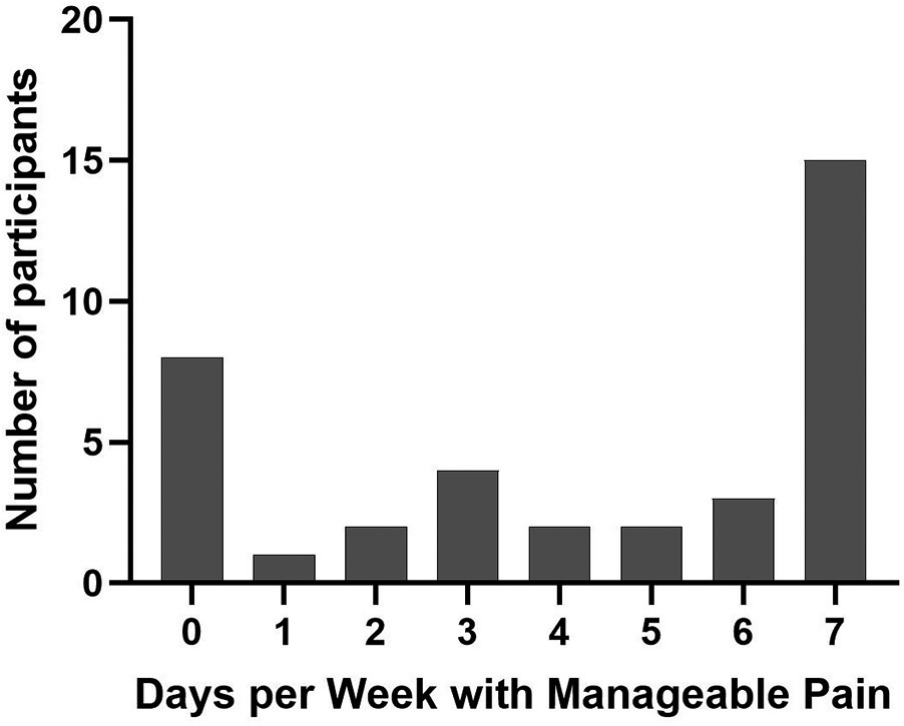
Frequency distribution of Participants by Days with Manageable Pain.

**Figure 2:**
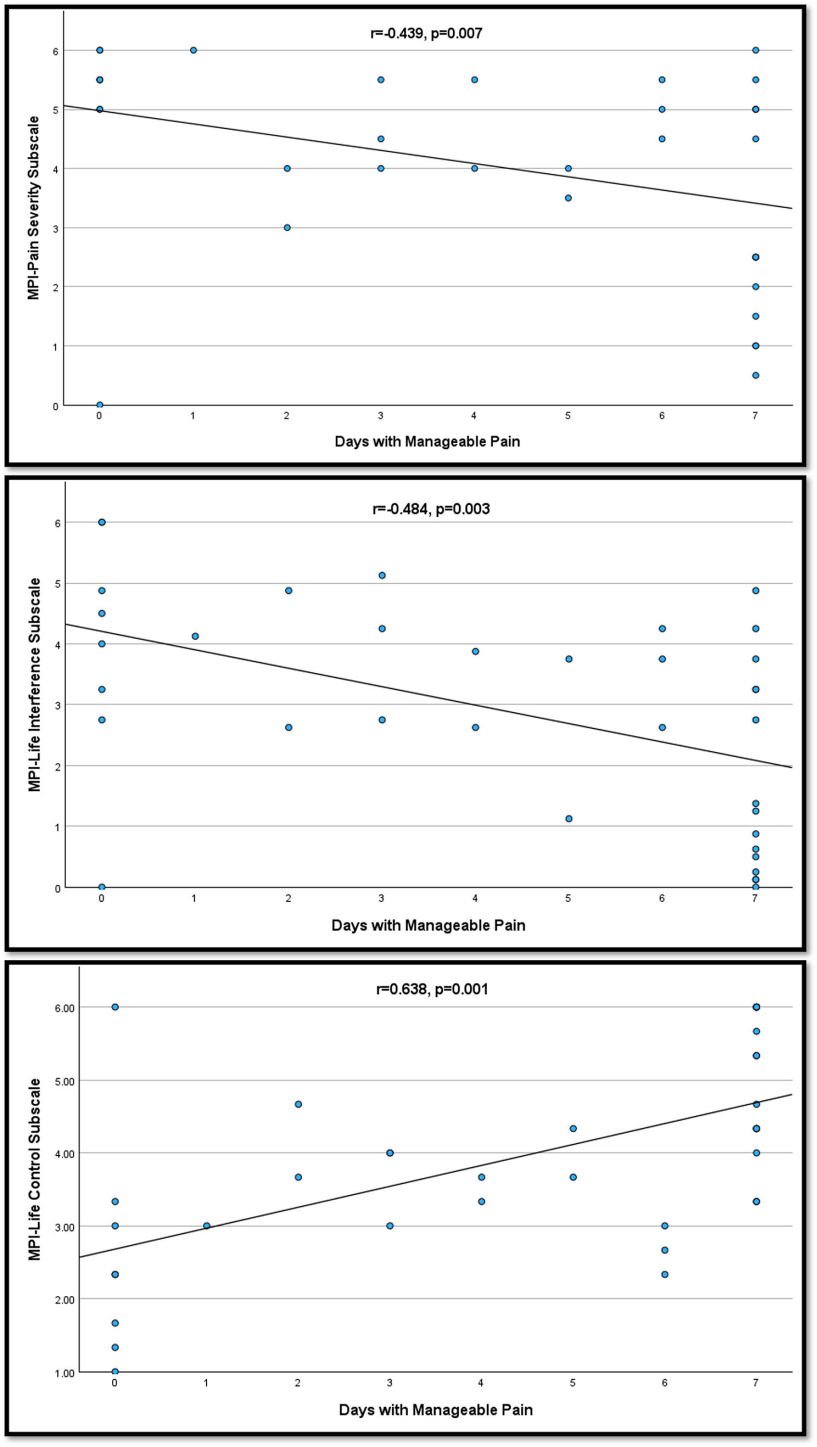
Correlation of Days with Manageable Pain with MPI Subscale Scores. MPI, multidimensional pain inventory; SCI, version.

The mean/median (standard deviation) of reported days per week with manageable pain (M-pain days) was 4.3/5.5 (2.9) (Figure 1). Additionally, M-pain days demonstrated moderate and significant correlations with MPI subscale scores for MPI-PS (*r* = −0.439, *p* = 0.007), MPI-LI (*r* = −0.484, *p* = 0.003), and MPI-LC (*r* = −0. 638, *p* = 0.001) (Figure 2). Additionally, M-pain days correlated with pain interference with activities (*r* = −0.5, *p* = 0.002) and difficulty dealing with pain (*r* = −0.439, *p* = 0.007). However, M-pain days was not correlated with the MPI-AD (*r* = −0.225, *p* = 0.187), mood interference (*r* = −0.287, *p* = 0.089), or sleep interference (*r* = −0.240, *p* = 0.159).

## Discussion

In this study, we explored the meaning of manageable pain in 36 people with chronic NP after SCI and associated measures of pain and psychosocial impact. As pain itself is known to be a very individual experience, so too were the factors that contributed to the perceived manageability of pain in this group. The qualitative analyses showed that “*manageable pain*” was often perceived as pain that is moderate and predictable, pain that can be controlled and dealt with, pain that can be ignored or tolerated, and pain that does not interfere with life.

In contrast, “*unmanageable pain*” was in some ways described as the opposite of manageable pain (i.e., pain that cannot be controlled and that hinders activity); it was also described as causing severe emotional distress. To control their pain, participants engaged in a wide variety of strategies, including medications, exercise, and physical activity, as well as cognitive/emotional coping strategies (including distraction). Importantly, most participants used a multimodal approach to control their pain, with varying combinations of the strategies listed above, which they developed through trial and error over time.

It is widely accepted that chronic pain is associated with numerous and different behavioral coping responses that may be adaptive or maladaptive, and that are dependent on individual resources (24–26). Specifically in those with SCI, adaptation and psychosocial impact have also been shown to be dependent on specific pain characteristics, including pain intensity, pain aggravation, electric quality, constancy, and distribution of pain (27). Further, repeated unsuccessful and frustrating attempts to control pain are known to actually exacerbate disability (28). It is important to note that all participants had NP with moderate to severe pain intensity (NPRS >3/10).

The present study also showed that the construct of “days with manageable pain” was supported by quantitative findings using validated questionnaires. Those who had more days with manageable pain also had lower pain severity, less pain interference with life and activities, greater life control, and reported lower levels of difficulty in dealing with their pain. It is possible that the number of days with manageable pain is influenced by the degree of pain acceptance. In another study of individuals with SCI and chronic pain, greater pain acceptance was related to better mood, social participation, and less pain interference, above and beyond the effects of same-day levels of pain intensity (29). Therefore, future studies should investigate the relationship between manageable pain and pain acceptance. However, it is also important to note that manageable pain may be influenced by a combination of personal factors such as coping skills, affective distress, resilience, nature, and the impact of pain on daily life (12, 30–35).

Finally, previous qualitative studies on NP in people with SCI have aimed to broadly describe the experiences of people in this population (13, 36–38), or to explore specific questions related to management strategies (39–41). This study was unique in that our aim was narrowly focused on describing specifically what makes pain manageable for people with NP after SCI and the associated pain and psychosocial impact characteristics. Another unique feature of this study was that it was conducted in South Florida, United States, which has a diverse population including Hispanic/Latino, Black, and White people and thus may provide a very different cultural setting from the locations in which prior qualitative studies were conducted. Specifically, other studies have been conducted in Canada (13), New Zealand (40), South Africa (38), and several countries across Europe [Netherlands (41), Sweden (42), United Kingdom (37), and Italy (36)]. Despite the geographical and cultural differences in participant cohorts across these studies, there are several important overlapping findings between these studies and ours. Collectively, these studies describe how participants with NP after SCI utilize trial and error to employ a wide range of coping strategies to manage their pain (13, 36, 38, 42). Additionally, distraction and learning to live with pain appear to be important coping strategies that cross racial, ethnic, and cultural boundaries in people with NP after SCI.

There are several limitations of this study worth discussing. All data collection was conducted in a top-ranked SCI research center embedded in a rehabilitation hospital, in an urban setting (Miami-Dade area). Thus, our sample may not be representative of all people with SCI. Additionally, all participants were enrolled in a multimodal pain management study when these interviews were conducted. Participants had also taken part in a series of educational sessions which may have impacted their perceptions of manageable pain. Therefore, the access and resources afforded to the participants in this study may not reflect the reality for many people with SCI, and this may have influenced our results. It is also likely that other factors not assessed in this study, including access to care and prior knowledge about pain, influence the ability to achieve days with manageable pain. Despite these limitations, this study achieved its goal and provides the field with the first qualitative description of the meaning of manageable pain in people with chronic NP after SCI.

Because NP is rarely completely ameliorated, a better understanding of what manageable pain means on an individual level and the factors that contribute to manageable pain is critical to improved quality of life after SCI. The findings of this study provide important data on an individual level. Our data also suggest that evaluating days with manageable pain may be meaningful in a clinical setting. Future studies should incorporate standardized measures of pain acceptance, coping strategies, and pain catastrophizing to improve understanding of the relationships between individual level factors and group level factors that contribute to manageable pain.

## Data Availability

The raw data supporting the conclusions of this article will be made available by the authors, without undue reservation.
