# Supplementary material for: The meaning of manageable neuropathic pain after SCI
Source: Front Pain Res (Lausanne). 2025 Jun 11;6:1540395. doi: 10.3389/fpain.2025.1540395 (PMC12187659; doi:10.3389/fpain.2025.1540395)
Supplement: Supplementary file 1 [file Table1.docx]

| **Managing pain** |
| --- |
| **How to control/manage pain** |
| 1. **Ignoring pain, using distraction** |
| **Participant 6:** But I always try to find a positive way of try to distract it from taking advantage of my life and pretty much controlling my life throughout. I know that it's there, but I try to black it out, pretty much.  And for me personally, I try to manage it. So I do the refresh of it and not try to avoid it. It's not avoidable because it's nothing that's not going to be there, but I just pretty much find a way to deal with it, to cope with it. |
| **Participant 9:** Mostly just ignore it. |
| **Participant 11:** So I just pay attention to how I'm feeling, and just let it pass with activity sometimes. |
| **Participant 35:** my pain levels aren't drastically high, so I tend to mostly just deal with it, have it in the background a lot of times and just carry on. I try to mostly just ignore it, essentially. |
| **Participant 37:** I put it in the mentality of, "Okay, is this pain hurting or is it bothering me?" Most of the times, it's just bothering me. It's not something that I cannot, how do I say it? It's not something that I cannot take all the time. Sometimes it gets really bad, but the moment I stop thinking about it or I get busy doing something else, it diminishes, it slows down. |
| 1. **Severity and situation dependent** |
| **Participant 8:** It's always manageable. It's just the form in which I manage it. If it's really intense pain, then I may have to use a medication or analgesic cream on it. If it's not that bad, it's just a matter of a mind over matter. |
| Participant 11: I mean, just take care of it early, don't wait too long, or be active and hopefully it'll pass. |
| **Participant 14:** For example, I have back pain. It mostly happens after a certain amount of time sitting in the chair. Once it reaches a certain limit, that's it. It's manageable because I lay down and I rest, and I'll take Advil |
| **Participant 27:** Sometimes exercise. Sometimes medication. Sometimes deep breathing techniques. There are options. Depends on what I'm going through like that, but those are the main things actually. |
| **Participant 35:** Once it starts climbing on the uncomfortable level, I have tried to rub different things, like on my arms, or for example, when it's on my butt, which is usually where I get the other pain, I usually just get off of it from sitting down. I just just get on my bed, lay on my side, just if I can stand or I can do something else, just relieve that pressure essentially. But mostly things like that. |
| 1. **Staying active, exercise** |
| **Participant 3:** Medication in a workout. I do stay active. Movement for my body, whatever my injuries are and whatever the signals are sent, the more I'm active, more warmed up I am, I can use my walker and push myself. There are moments that my legs trip. I have to slow down, not to rush. It's not easy pushing the walker. I cannot stand completely straight, so I'm leaned over, but I'm walking, which is an important part.  I've been with them like five, six years and that stretching and exercise works better than any other therapy for me. |
| **Participant 18:** At the beginning, I have to tolerate the pain that I was having at that moment, still have. But it seems that playing the sport, it really mentally and physically minimize the pain. I've been playing a lot of sports.  Yeah, because mental, I have to concentrate in a specific kind of exercise, let's say, and that help me out to get to not think about the pain, in a way. When I'm sweating, I feel good physically. I'm thinking,"Oh man, I'm sweating now. I'm going to kind of have a good physiques, good. I going to develop good muscle tone, everything." And on top of that, I'm kind of very active. So that really, in a way, it really, to me, minimize the pain. That's how I can put it. |
| **Participant 20:** The manage pain... so when you moving right, when you got the exercise, when you have the exercise, your pain is not the same when you don't have the exercise. Now, when you manage your pain, when you manage for exercise, your pain's coming better now. |
| **Participant 32:** And then when I got to my mother's house, I had to learn. I was taking some heavy pills that time, but then my mother didn't want me to take them anymore, because of the addiction. So I had to learn other things like exercise, read a little bit about it, even though I don't remember the words. |
| **Participant 33:** I just move my neck and then I have a little massager. And then I just put it on the side of my neck and it relieves it for a little bit. Then I got to go to the other side and do the same thing |
| 1. **Using medication** |
| **Participant 3:** the doctor, they told me to switch it to Tylenol, not always take ibuprofen. I used to take two a day and I reduced it to one, but even that would bother me. And then I said, "What do I do? It's not helping me." Try Tylenol. It's not the same side effects. Tramadol, I take, instead of two, a day, I take one a day too, but it's giving constipation and I take something for constipation. And the diet, helps to have more liquidity diets and so forth anyway.  So I switching the medication, not sticking just to the prescription to take three a day, two a day, something like that. And I accept the pain on daily basis. |
| **Participant 4:** I take oxycodone, tramadol, I think. Blood pressure, blood thinners. Lot of mine is like vitamins. I had some for the spasms. I take a pain for that... I mean, pills for that. Like I say, most of mine like vitamins.  I'm accustomed to it, so I just deal with it. I ain't going to lie, I smoke my little weed. Weed helps ease the pain too. It helps a whole hell of a lot. |
| **Participant 6:** I pretty much just try to take my medication with it. And once I take my medication with it... Because without the medication... if I'm not going to take the medication, it's [inaudible 00:02:05]. That's not much of a different. But that's a big difference when you don't take a medication than when you do take your medication. And then once you take your medication... It's there. It never goes away, with the medication or whatever. With the medication, it's just a way to cope with it. |
| **Participant 11:** but I honestly just smoke weed for my pain if it gets to that point. I'm not a person that takes pills or anything of the nature. |
| **Participant 12:** I'd say pretty helpful because I was just really struggling and the pain that I feel, probably everybody's different, but my pain is if you had an iron that your iron your clothes with. And if you were sticking it on your skin and there's a five centimeter area around my entire breasts. It goes for my breasts all the way around my back. Now, since I've been using the medication, there are spots now that really hurts and one is my left nipple and then the right side of my back. And it really feels like someone has an iron they're pressing it hot. And so, like I said before I started taking the medication, like an inner tube that was just hot and burning. And then now, like I said, it's mostly the left nipple and the right back.  And I put Diclofenac on it and I put Lidocaine patches sometimes. And this last week, the ice packs really helped a lot because that was really burning and the ice just made it feel much better. It actually sort of froze the pain |
| **Participant 13:** The dream cream. I feel like it helped me manage my pain a little bit better. |
| **Participant 21:** the morphine and the marijuana kind of helps out a little bit |
| **Participant 24:**  only way I can get lower levels of pain is when I take these larger amounts of narcotics, which I don't like. And then I'm a zombie, so talking to my spouse or anything like that, I forget half or all of what was said at times she tells me, which if I tried to translate that into any kind of work environment, just not even in the realm of possibility. So maybe a medication that doesn't have so many side effects. |
| **Participant 28:** taking meds. That's the only way right now that I'm able to control it and go out and do whatever I need to get done. You know, like maybe doctor's appointments, or maybe even going to the rehab center. I have to take a gabapentin and a clonazepam. |
| **Participant 32:** So I take TOC. It helps a little bit to a certain point. All the practice that I was mentioning you, it helps me to a certain point. Right now I feel a little bit bothered, but I could hang on. |
| **Participant 36:** Right now I'm just using marijuana, gabapentin, and duloxetine. |
| 1. **Using multiple approaches** |
| **Participant 1:** For example, this Saturday was my granddaughter's one year old, but I woke up with tremendous pain, one of the worst, and I had to take some marijuana cream and then I had to take Motrin to see if I could manage my pain. Where I do manage my pain more than when I just told you about it is that I redirect my pain in my thoughts. I just don't think about it. I try to just focus on the event and everything else. I blank out on the pain. |
| **Participant 2:** with medicine or with exercise or with my hand braces or stretching or that there's something I can do about it.  But the worst part for me, for example, is sitting in a car. Sometimes we have to pull over just for me to get out and stretch. I have to pressure my hands down and put a lot of weight, like weight bearing on my arms to get rid of the pain, so that's when it's harder to manage. Just a medicine, a pain killer would be great so I can go through a 45-minute car ride. And then I have to stop just to stretch or bring my hand braces with me because they actually have reduced some of the pain. But sometimes I don't think about bringing them. And they're just so ugly, I don't want them. And so I don't like to take them places with me. People look at them weird. |
| **Participant 11:** but I honestly just smoke weed for my pain if it gets to that point. I'm not a person that takes pills or anything of the nature. So it's no other way to deal with it, but just to pay attention to how you've been going about with heat, cold, difference in how you're sitting and stuff like that. |
| **Participant 17:** To manage it would be to be on some type of medication regimen, if that's what you're on. Or some exercise routines. I tend not to think about pain that often. So I feel like the more you think about it and you spit on it, you manifest it, you're bringing it into your life. So I'm very intuitive and I just try to balance it like that. I also, honestly, I don't know if I can say this, but in all honesty, I smoke a lot of weed too. And I feel like that also helps me manage my pain. |
| **Participant 21:** thought everybody goes through their own process when you're spinal cord injury or something like that. But, yeah, yeah, yeah. It's like when you first get into a game you don't know the game, but when you play a couple now you know the map. The game seems smaller now. |
| **Participant 24:** Tools being medication, stretches, exercise, rests, the list could go on, and those tools help when I get in extra pain. |
| **Participant 32:**  Okay, let me try to be specific with you. I do a lot of stuff. I do a lot of reading, I do a lot of stretching, I do a lot of exercise. I do a lot of massage, I massage myself. I do a lot of stretching. I do some breathing techniques, meditation. I sleep pretty good, even though I have to wake up and shift. And sometimes right now because it is kind of cool, the pain in my left side, it bothers a little bit, but that's normal already. That's something that I'm really used to. |
| **Participant 37:** I meditate, I work out, I try to eat as healthy as I can, except on the weekends because I like garbage, but I try to maintain a good, healthy body, mind type of set. |
| **Participant 38:** Different methods, whether it be taking a little bit of Tylenol, whether it be doing physical therapy or occupational therapy or doing some type of exercises or swimming, some meditation, breathing techniques, things of that nature. |
| **What is manageable pain?** |
| 1. **Manageable pain characteristics** |
| **Participant 7:** When it's manageable, it's not extreme. It's just not extreme, I guess  It's not aching or burning.  It depends on what areas. Usually, if one area's hurting very badly, then another area's not as severe, but it all depends. Sometimes, maybe more than one area is hurting at once or maybe it's not.  Speaker 1:  So manageable pain is related to the other pains too? I mean, to all the different pains that you have.  Speaker 2:  Yeah. Correct. |
| **Participant 12:** Ideally, it would be zero pain, but that's pretty hard. That's pretty hard to get. Once in a while, I do get that. I think it's mostly when I'm being distracted, but manageable pain. Let's see. I think probably if you're using that scale, I think a four or five would be manageable. |
| **Participant 13:** If it is not so intense |
| **Participant 24:** It would be more consistent pain rather than the spikes, which I mentioned and I think that's the main one.  Steady state and lower levels of pain. |
| 1. **Pain that can be controlled** |
| **Participant 1:** but how to turn off the pain and turn back and then after where you don't need it, let the pain come back up. That's what managed pain means to me, that I can, if I'm going to this event that I went to last Saturday, that I can do something to manage that pain beforehand, not just redirect my thought and not think about it, but manage it without medicine, the medication. That I would love to do. |
| **Participant 2:** That I can control it, that I can take care of it. |
| **Participant 6:** To me, right now, the manageable pain is pretty much as a way to find a way to deal with the pain that I have, which is always there. |
| **Participant 8:** Pain that you can ... what's the word I'm looking for ... relieved by either medication or exercise or meditation or any type of form where you can manage it where it's not overwhelming or controlling your life in a negative way. |
| **Participant 9:** that you have a way of controlling it. |
| **Participant 13:** Pain that you can manage. |
| **Participant 17:** it means being able to manage it, to control it, to take care of your pain when you ... Manageable pain to be able to manage it. |
| **Participant 21:** It's just finding a way to manage pain. Sometimes finding ways like toughening out or, I guess, researching with your pain management to see what can help out or anything like that. But yeah, usually just seeing whatever you can do to manage that, push through. That's what it means to me, some of it. |
| **Participant 22:** The relief that you get with the one that you're able to manage. |
| **Participant 24:** Particularly when I get dopey, it helps with the pain, but then I'm no good for anything else. So to be manageable, I'd like to think I can accomplish something on a monthly basis. I used to be very accomplished in my professional career and now I forget basic things in life, which I attribute to heavier medication doses. |
| **Participant 25:** It's pain that I could somewhat control. A pain that I could possibly take some type of medication or do some type of non-medicated therapy that could actually help alleviate or control the pain to some degree. |
| **Participant 30:** Manageable mean that I take medicine. |
| **Participant 33:** Well, it's manageable because when I stretch my leg and then, well, it's really manageable. Yeah.  That I can manage it. I can handle it or do like I'm doing, I stretch my legs so I can relieve my pain.  That I can manage it. I can handle it or do like I'm doing, I stretch my legs so I can relieve my pain.  I just move my neck and then I have a little massager. And then I just put it on the side of my neck and it relieves it for a little bit. Then I got to go to the other side and do the same thing. But I still do, I do |
| **Participant 36:** manageable would be being able to control it |
| **Participant 38:** What it means to me is how I could manage and tolerate the pain that I have at this moment and what I can do to help alleviate myself from feeling so much pain on a daily basis.  Manageable pain is something that I could tolerate and control |
| 1. **Pain that can be ignored or tolerated** |
| **Participant 12:** it's something that I could force myself to deal with for a little period of the time. |
| **Participant 15:** Manageable pain is pain that doesn't necessarily stop me from doing what I need to do, whether it's [inaudible 00:01:21] or being able to handle daily tasks without being shutdown, where it's too much in pain where all I want do is be shutdown for the day. So anything that's... not necessarily no pain, but I can deal with it. It's bearable. |
| **Participant 16:** Well, it's abdominal pain, and you might take some medication. Then, okay, that's helping. Some of them do, but some of them may be not effective as some others. That's just how I feel, and that's the way you manage pain. Or it could be your brain; your mind. Sometimes, just trying to think about something else or do something else, just to ignore that pain. |
| **Participant 18:** I used to be able to control the pain, either mentally or physically, by doing something physically or visualizing something. And also by controlling yourself, explaining that, I mean, this is not really going to bother me if it is a moderate pain. I'm not in the stage that I have a tremendous pain I cannot tolerate. But yeah, basically that's what I think. |
| **Participant 19:** The ability to forget about it. So it could still be there, but you just aren't consciously thinking about it. |
| **Participant 22:** Well, I have different points. The pain that you could manage it, the pain that you would be able to take something or do something and it would go away and you would have no pain. But unfortunately, that's not our reality. So, in this case, we are just able to shift and make it in a way where you can live with it.  Yeah, because in my case, if it doesn't go away, I'm sorry. If it doesn't go away, I have to learn how to live with it. |
| **Participant 23:** Manageable pain. Basically just any pain that doesn't hurt too much, and I can almost just kind of forget about it, even though it hurts really bad, if that at all makes sense.  I can forget about it until I'm reminded of it again and it's still there. |
| **Participant 27:** Just dealing with the shit I got to deal with to get through it. It's like finding whatever's copacetic to make it work for me. |
| **Participant 30:** my pain is less. I can be comfortable. I can sleep well. |
| **Participant 32:** Learning how to control your injury. Injury, or some type of suffering, pain that you're going through. Learn how to live with it.  **Participant 33:** I go outside, I go to the backyard, I work out, I stretch out there in the back. But I just forget about the pain. |
| **Participant 37:** I think I've had pain, regardless. I don't think in any way that you can manage pain, but you can definitely not think about it. I think it takes practice to not think about it. But yeah, I don't think there's anything, there's no such thing as manageable pain. I just think that you become very good at not thinking about it and that's what I do. I just don't think about my pain and then I don't have pain. I mean, I still have pain, but I don't let it get the best of me in that sense, if that makes sense? |
| **c. Pain that does not interfere with my daily life activities** |
| **Participant 1:** That I'm able to manage my pain when I'm going to an event, that I'm going to see family, going to a restaurant.  That I'm able to manage my pain when I'm going to an event, that I'm going to see family, going to a restaurant. |
| **Participant 2:** So it's manageable when I'm able to stand up and stretch, especially because I am able to stand. I'm more mobile than everyone with the spinal cord injury. |
| **Participant 10:** It means that I can go out for a little bit throughout the day, and that the pain isn't stopping me from living my daily life. I always have pain, but the manageable pain is that I can go to the store for an hour or go sit in the car, or go out and do something like that, then go back home. |
| **Participant 14:** Pain you could live with; maybe just continue your daily activities. I think that's what manageable pain means to me. |
| **Participant 15:** I can still do what I need to do. |
| l **Participant 19:** pain that isn't bothersome so I can continue living my life. That like it's there, but it's not going to affect my day-to-day |
| **Participant 24:** Elusive. It's been almost four years since my injury. Don't ever feel like I've managed it. Everything in life is set up to try to manage it, but manageable pain, for me, is not what I have. It would mean I could do more activity without the big spikes in pain that I get every day, or inexplicable spikes that happen when there's no activity, that I don't understand. So manageable, that's a tough question, because I haven't experienced that. |
| **Participant 26:** I would say something, I could still get up and still do my daily tasks without ... Well, I will complain but I'm still doing it. That's manageable. |
| **Participant 27:** So I can focus through the day. In other terms of manageable is being able to function at a level that's not too unbearable for them. |
| **Participant 28:** That I'm able to go out and do whatever I need to do.  Let's say you got to go and do something, you got to do something around the house, pain is management of that you can go and do that without say cancel. |
| **Participant 29:** It means basically that something that you can go through the day without have to say, well cut back anything you have to do. |
| **Participant 32:** Well that's when, let's say when my body is already tired, like that's it. No more. It doesn't have the strength. I have to take something stronger to be able get drowsy. |
| **Participant 35:** Just pain that I can deal with and just carry along doing things while still being in pain, but still being able to do things, I guess.  Live my life type of stuff. |
| **Participant 36:** I can manage my pain and go throughout my day without having to deal with the pain. |
| **What makes pain unmanageable?** |
| 1. **Affects mood** |
| **Participant 4:** So I talked to him a lot, and he explained the way how he been dealing with it. He say he still have his pain and all that, but he here. You can't... He told me, "Don't question God, just live." Just live like if he was walking. Because, I'll tell you, sometime I want to give up. I have my days where I don't want to be bothered. I have plenty days where I just sit there and cry. Why I cry? I don't know. But I just be boo-hoo crying. |
| **Participant 6:** If you don't manage it, pretty much, you don't feel like doing pretty much nothing at all. You are angry after work. |
| **Participant 16:** Yeah, it's really strong. It's [inaudible 00:02:49]. I would rather be like, "Oh, man. I don't want to go through this." I would probably rather be dead, than deal with something like this. It's killing you. Softly. Maybe not saying anything, but it's killing you. Even if nobody not see it; who can see it? You're the only one that knows, because you don't want to feel it. It's not manageable. That's like waiting for the [inaudible 00:03:17]. They on, they know you'll learn. That's why I like the educational things. |
| 1. **Cannot be controlled or tolerated** |
| **Participant 2:** If I have to hold a position, like to stretch it, and then I go right back to laying down and it's right back to being as severe as it was before I stretched, that would be when it's not manageable. [inaudible 00:01:24] everything manageable, yeah.    So that's when it's less manageable. That's when you wish you just had just a pill that could just take it away for the moment, something that's not going to put you to sleep. |
| **Participant 9:** Sometimes I have a throbbing in pain that's not manageable at all, it feels like it's deep down in the muscle.  It's almost like a needle stuck in there. It's not often, but sometimes. |
| **Participant 14:** but if I don't lay down, then it becomes a problem because if I push it past that limit, then I'm down for the next two or three days. |
| **Participant 17:** not doing any of those things above, I guess. |
| **Participant 18:** pain that really cannot control. For example, if I am in my bedroom, if there is no any other visual or physical activities to do, I'm just by myself, very quiet. In a very quiet environment, I would feel the pain sitting there or in my bed doing nothing. So that's where, unfortunately, I can experience the pain and nothing to do about it. That's how I can describe it. |
| **Participant 22:** The other one is just rough. Like, okay, I guess I have to live with it. |
| **Participant 36:** unmanageable is not being able to control it and it just having a mind of its own like it does. |
| **Participant 38:** unmanageable pain is something that I just won't be able to tolerate and control and it's just unbearable. Yeah. |
| 1. **Hinders activity** |
| **Participant 1:** Yes. Yes, especially when I'm going to a dinner or I'm going to a meeting. When I'm going to an expo, when I'm going out with my family, business, one of the main things business is, how's my pain going to be while I'm sitting there in a seven-hour conference? About two weeks ago, I went to a conference here in Miami for my business. I had to. I didn't go to the whole 8:00 AM to 5:00 PM because I wouldn't be able to manage that pain that long. I knew that I could manage that pain, let's say, from 2:00 to, actually, I stayed there from 2:00 to 8:00, but I wouldn't be able to do that 12 hours, just sitting there. |
| **Participant 35:** where I really can't be doing most things, having a hard time getting along with people and not wanting to go out. Just being basically, I guess you could say, incapacitated, disabled from the pain |
